# Discrepancies Between Perceptions of the Parent–Adolescent Relationship and Early Adolescent Depressive Symptoms: An Illustration of Polynomial Regression Analysis

**DOI:** 10.1007/s10964-016-0503-5

**Published:** 2016-05-26

**Authors:** S. A. Nelemans, S. J. T. Branje, W. W. Hale, L. Goossens, H. M. Koot, A. J. Oldehinkel, W. H. J. Meeus

**Affiliations:** 1 Research Centre Adolescent Development, Utrecht University, Utrecht, The Netherlands; 2 Department of School Psychology and Child and Adolescent Development, KU Leuven, Tiensestraat 102 – bus 3717, 3000 Leuven, Belgium; 3 Department of Developmental Psychology, Vrije Universiteit Amsterdam, Amsterdam, The Netherlands; 4 University of Groningen, University Medical Center Groningen, Groningen, The Netherlands; 5 Department of Developmental Psychology, Tilburg University, Tilburg, The Netherlands

**Keywords:** Early adolescence, Depressive symptoms, Multiple informants, Parent–adolescent discrepancies, Polynomial regression analysis, Difference scores

## Abstract

Adolescence is a critical period for the development of depressive symptoms. Lower quality of the parent–adolescent relationship has been consistently associated with higher adolescent depressive symptoms, but *discrepancies* in perceptions of parents and adolescents regarding the quality of their relationship may be particularly important to consider. In the present study, we therefore examined how discrepancies in parents’ and adolescents’ perceptions of the parent–adolescent relationship were associated with early adolescent depressive symptoms, both concurrently and longitudinally over a 1-year period. Our sample consisted of 497 Dutch adolescents (57 % boys, *M*_age_ = 13.03 years), residing in the western and central regions of the Netherlands, and their mothers and fathers, who all completed several questionnaires on two occasions with a 1-year interval. Adolescents reported on depressive symptoms and all informants reported on levels of negative interaction in the parent–adolescent relationship. Results from polynomial regression analyses including interaction terms between informants’ perceptions, which have recently been proposed as more valid tests of hypotheses involving informant discrepancies than difference scores, suggested the highest adolescent depressive symptoms when both the mother and the adolescent reported high negative interaction, and when the adolescent reported high but the father reported low negative interaction. This pattern of findings underscores the need for a more sophisticated methodology such as polynomial regression analysis including tests of moderation, rather than the use of difference scores, which can adequately address both congruence and discrepancies in perceptions of adolescents and mothers/fathers of the parent–adolescent relationship in detail. Such an analysis can contribute to a more comprehensive understanding of risk factors for early adolescent depressive symptoms.

## Introduction

Adolescence is a critical period for the development of internalizing problems, particularly depressive symptoms. In adolescence, depressive symptoms are quite common (Kessler et al. 2012; Merikangas et al. 2010) and once these symptoms emerge, they remain quite stable over time (Holsen et al. 2000; Kessler et al. 2012) and appear to have a substantial impact on adolescents’ concurrent and later psychosocial functioning (Birmaher et al. 1996). Therefore, it is important to understand factors that may increase risk for the development of depressive symptoms in early adolescence. One of the factors that has been consistently associated with higher adolescent depressive symptoms is problems in the parent–adolescent relationship (Hale et al. 2008; Joiner and Coyne 1999; McLeod et al. 2007; Nelemans et al. 2014; Rudolph 2009). At the same time, adolescents and parents often perceive their relationship quite differently, and some authors have suggested that differences in these perceptions may be particularly important to consider as a risk factor for the development of adolescent depressive symptoms. Therefore, in the present study, we aimed to examine how discrepancies in adolescents’ and parents’ perceptions of their relationship were associated with early adolescent depressive symptoms.

### Theoretical Perspectives on Informant Discrepancies

In both clinical practice and research on family functioning and child adjustment, the inclusion of multiple informants has become highly desirable. Including multiple informants is important because studies have demonstrated that various viewpoints, specifically those of the child and the parent, are not mutually exclusive and may each offer valid information (e.g., De Los Reyes and Kazdin 2004, 2005; De Los Reyes et al. 2015). Yet, these multiple viewpoints often bring with them disagreements among informants’ reports on the same construct of interest, generally labeled as informant discrepancies. For example, the extensive line of research on parent–adolescent discrepancies in reports of adolescent functioning has yielded a robust set of findings, suggesting low-to-moderate correspondence between parent and adolescent reports of adolescent problem behavior (Achenbach et al. 1987; De Los Reyes et al. 2015). It is important to realize that these discrepancies among informants’ reports can substantially impact researchers’ interpretations of research findings. Specifically, researchers may obtain different findings, and may therefore reach different conclusions, depending on which informant they rely on.

But what does it mean when informants disagree? Previously, discrepant views between informants have been mainly conceptualized as resulting from informants’ unique perspectives (including reporter bias) and measurement error. This view has resulted in efforts to search for optimal ways of integrating reports from multiple informants and combining these reports into a single estimate as the best possible approximation of a construct, which would be a more reliable index than any single informant report (for a discussion, see Kraemer et al. 2003). However, our knowledge of what informant discrepancies represent has been steeply increasing over the past two decades (De Los Reyes 2011; De Los Reyes and Kazdin 2004, 2005; De Los Reyes et al. 2015). This growing body of knowledge has led to an increased appreciation that discrepant views between informants yield meaningful information and thereby are informative in and of themselves. For example, informant discrepancies may yield meaningful information on different contexts in which behaviors are expressed, and may point to systematic differences between informants in how they observe and interpret behavior (De Los Reyes and Kazdin 2005).

Theoretically, informant discrepancies may be understood from models that emphasize the importance of a so-called *fit*, or match, between individuals and their environment. First, the original concept of *goodness of fit* was introduced with respect to the match, or mismatch, between a child’s temperament and the parental environment (Thomas and Chess 1977), which was further elaborated on by Lerner and colleagues (Lerner 1985; Lerner et al. 1986). From this perspective, low parent–child fit is assumed to negatively affect child development and adjustment. Specifically, the extent to which children can meet the demands of a parent, or a particular setting, (i.e., goodness of fit) determines how significant others such as parents react to the child, which feeds back to affect child development and adjustment. These parental demands may take the form of attitudes, values, or expectations, to which the child must “fit” his or her behavior. Children who do not fit or fall short of parents’ demands are therefore at risk for maladaptive outcomes (Lerner et al. 1986).

Lerner (1985) extended the concept of goodness of fit to adolescent development and adjustment, particularly during early adolescence, as this developmental period appears to be characterized by a wide array of new and unique psychological and social demands that need to be negotiated, including major developmental changes in the parent–adolescent relationship (Laursen and Collins 2009). These changes in the parent–adolescent relationship, together with the young adolescent’s strive for separation, individuation, and increased autonomy from early adolescence onwards, go together with substantial changes in social and contextual demands. These changing demands, in turn, make discrepancies between adolescents’ and parents’ perceptions of their relationship especially likely to occur in this developmental phase. Hence, early adolescence is a particularly important developmental period to study parent–adolescent discrepancies, as these may substantially increase the risk for the development of adolescent depressive symptoms during an already critical period for the development of these symptoms.

Second, from a similar line of reasoning as goodness of fit, *stage*-*environment fit theory* (Eccles et al. 1993) also specifies that adolescence, and early adolescence in particular, is characterized by a developmental mismatch between the needs of developing adolescents and the opportunities provided by their social environments, including the parent–adolescent relationship. The larger this developmental mismatch, or the lower the “fit” between the developing adolescent and social (i.e., their parents’) demands, the larger the discrepancies between adolescents’ and parents’ perceptions of their relationship are likely to be. These discrepancies, even more so than adolescents’ increasing negative view of the parent–adolescent relationship from early to mid-adolescence (De Goede et al. 2009), might lead to adolescents feeling misunderstood and thereby to higher adolescent depressive symptoms. This theoretical model also highlights early adolescence as particularly “problematic” with respect to stage-environment fit, further stressing the need to study this specific developmental period with respect to parent–adolescent discrepancies and their impact on adolescent depressive symptoms.

The aforementioned concepts of person-environment fit directly link to informant discrepancies, in a way that lower discrepancies between adolescents’ and parents’ perceptions of their relationship may be reflective of better fit between them and may thereby be associated with healthy adolescent development. By contrast, when adolescents’ characteristics or needs do not match with their parents’ demands, this lack of fit may result in discrepancies between adolescents’ and parents’ perceptions of their relationship and this reflection of incompatibility between adolescents and parents may be associated with higher adolescent depressive symptoms. A link between poorness of fit between the child and the parental environment and depressive symptom development has also been put forward by Thomas and Chess (1977), when they linked poorness of fit to crystallization of a negative, denigrated self-evaluation in children, which may be considered as a precursor for, or an aspect of, depression.

### Methodological Considerations Associated with Informant Discrepancies

Goodness of fit hypotheses have been generally tested using discrepancy or difference scores, with larger discrepancies reflecting worse fit. Interestingly, the most widely used approach in studies on informant discrepancies also relies on the computation of difference scores (i.e., subtracting one informant’s report from another informant’s report). In line with goodness of fit expectations, findings from previous studies using difference scores as indicators of informant discrepancies suggest that larger discrepancies in parents’ and adolescents’ views of the parent–child relationship, or parenting, are associated with worse adolescent outcomes (De Los Reyes et al. 2010; Stuart and Jose 2012), including adolescent depressive symptoms (Guion et al. 2009; Juang et al. 2007; Sher-Censor et al. 2011). However, there are various ways in which difference scores can be calculated and the specific calculation substantially impacts its meaning (Edwards 1994; Laird and De Los Reyes 2013). Moreover, Laird and De Los Reyes (2013) have recently criticized the use of difference scores as test of hypotheses involving informant discrepancies, as several issues challenge their validity (in addition to numerous other methodological problems from which difference scores suffer; see also Edwards 1994, 2002).

As a superior alternative to the analysis of informant discrepancies—one that reduces or avoids the limitations of difference scores—Laird and De Los Reyes (2013) have suggested the use of polynomial regression analyses including tests of moderation (in line with Edwards 1994, 2002; Laird and Weems 2011). Statistical tests of moderation, including an interaction term between two informants’ reports (e.g., mother-report multiplied by adolescent-report), allow for conclusions regarding the importance of the unique combination of two informants’ reports, above-and-beyond the potential independent contributions of the individual informants’ reports. Such interaction terms provide key tests of informant discrepancies, by directly testing whether associations between, for example, adolescent-reported quality of the parent–adolescent relationship and early adolescent depressive symptoms vary as a function of parent-reported quality of this relationship. Importantly, in a recent cross-sectional study such interaction terms have been found to show criterion validity as indirect measures of parent–adolescent discrepancies in reports of parenting (De Los Reyes et al. 2013). Yet, as most of the existing work on parent–adolescent discrepancies and adolescent adjustment, including adolescent depressive symptoms, has relied on the use of difference scores as indicators of informant discrepancies, it remains unclear to what degree substantive conclusions based on these results are supported by results from polynomial regression analyses.

### Discrepancies in Reports of the Parent–Adolescent Relationship and Early Adolescent Depressive Symptoms

Whereas most previous studies on discrepancies in parents’ and adolescents’ views of their relationship and adolescent depressive symptoms have been cross-sectional in nature and have used difference score methods as indicator of parent–adolescent discrepancies (e.g., Guion et al. 2009; Juang et al. 2007; Sher-Censor et al. 2011), one longitudinal investigation by Laird and De Los Reyes (2013) has used polynomial regression analyses including tests of moderation to examine whether adolescent-parent discrepancies in views of the parent–adolescent relationship predicted later adolescent depressive symptoms. This study found that interactions between mother and adolescent reports of conflict and acceptance in the mother–adolescent relationship significantly predicted adolescent depressive symptoms. Specifically, *congruence*, but not *discrepancies,* between mother and adolescent reports in low levels of conflict and high levels of acceptance was associated with the lowest levels of adolescent depressive symptoms. By directly comparing these results to those from difference score analyses, as well as explicitly testing some of the mathematical assumptions underlying difference scores, these authors concluded that polynomial regression analyses including tests of moderation are more valid for examining hypotheses involving informant discrepancies and that researchers should avoid using difference scores, as they are likely to produce inaccurate conclusions. Because studies using such polynomial regression analyses to test hypotheses involving informant discrepancies are still scarce, particularly in the field of parent–adolescent discrepancies and adolescent depressive symptoms, more research is necessary to examine how results obtained from different methods relate to one another and to better understand how empirical conclusions may be affected by the particular method selected.

Furthermore, family systems theory argues for the importance of distinguishing between the mother–adolescent and father–adolescent relationship in their association with adolescent depressive symptoms, as these relationships represent distinct subsystems within the family (Minuchin 1985; Restifo and Bögels 2009). Most existing research has focused on the mother–adolescent relationship in association with adolescent depressive symptoms or has not distinguished between the mother–adolescent and father–adolescent relationship (by asking adolescents about their parents in general), and the few studies that have examined associations for the father–adolescent relationship have produced mixed findings. For example, some studies suggest that problems in both the mother–adolescent and the father–adolescent relationship are associated with adolescent depressive symptoms (e.g., Marmorstein and Iacono 2004; Vazsonyi and Belliston 2006), whereas others have found no such associations concerning the father–adolescent relationship (e.g., Margolese et al. 2005). At the same time, we know little of how *discrepancies* in the mother–adolescent and the father–adolescent relationships may, or may not, be differentially associated with early adolescent depressive symptoms.

In addition to parents’ gender, adolescents’ gender may also be important to consider as a potential moderator of associations between discrepancies in the mother–adolescent and father–adolescent relationships and adolescent depressive symptoms. Interpersonal theories of depression (Rudolph 2009) suggest that girls show greater vulnerability to relationship disturbances than boys, perceive interpersonal problems as more stressful than boys, and show higher levels of depressive symptoms in the face of relationship disturbances. Hence, problems in both the mother–adolescent and the father–adolescent relationship may be expected to be more strongly associated with depressive symptoms for early adolescent girls than boys. Yet, no studies have previously examined whether adolescent gender moderates associations between discrepancies in the mother–adolescent and the father–adolescent relationships and early adolescent depressive symptoms.

## The Present Study

In the present multi-informant, 2-year longitudinal community study, we aimed to examine how discrepancies in parents’ and adolescents’ perceptions of the parent–adolescent relationship were associated with early adolescent depressive symptoms, both concurrently and longitudinally over a 1-year period. We focused on one specific dimension of the parent–adolescent relationship that has been consistently associated with adolescent depressive symptoms (Hale et al. 2008; McLeod et al. 2007; Nelemans et al. 2014) and is central to several theories on adolescent depression (e.g., Joiner and Coyne 1999; Rudolph 2009), that is, the negative and conflictual aspect of the parent–adolescent relationship. Furthermore, in line with family systems theory, we distinguished between discrepancies in the mother–adolescent and the father–adolescent relationship in association with early adolescent depressive symptoms and considered adolescent gender as a potential moderator of these associations.

This study extends prior research in several ways. First, in line with family systems theory (Restifo and Bögels 2009) and recent calls to study the mother–adolescent and father–adolescent relationship separately when examining associations with adolescent depressive symptoms (Brumariu and Kerns 2010), we distinguished between perceptions of the mother–adolescent and the father–adolescent relationship to examine potential differential associations with early adolescent depressive symptoms. Second, this study specifically focused on the early adolescent period, just after the transition from elementary to secondary school. This developmental period is particularly salient to our study aim, not only because marked changes in the parent–adolescent relationship are taking place in early adolescence that are associated with increasing parent–adolescent disagreement about parenting behaviors (Laursen and Collins 2009), but also because early adolescence is a vulnerable period for the development of depressive symptoms (Cole et al. 2002; Rudolph 2009) and a developmental period in which decreased person-environment fit is especially likely to occur (Eccles et al. 1993; Lerner 1985). Third, we followed recent suggestions (Laird and De Los Reyes 2013; Laird and Weems 2011; see also Edwards 1994, 2002) that have recommended using polynomial regression analyses including tests of moderation, rather than the use of difference scores, as tests of hypotheses involving informant discrepancies. Fourth and finally, we examined all associations with early adolescent depressive symptoms both concurrently and longitudinally over a 1-year period, controlling for adolescents’ earlier depressive symptoms. In light of the relative stability of depressive symptoms over time (Holsen et al. 2000; Kessler et al. 2012), it is crucial to check whether discrepancies in perceptions of the parent–adolescent relationship have any additional value above-and-beyond the relative stability of depressive symptoms over time. Yet, most previous studies have been mainly cross-sectional or longitudinal without controlling for earlier depressive symptom levels.

Using interaction terms in polynomial regression analysis (Laird and De Los Reyes 2013), we tested whether associations between higher (or lower) adolescent reports of negative interaction in the parent–adolescent relationship and early adolescent depressive symptoms varied as a function of higher (or lower) mother/father reports of negative interaction. Vice versa, we tested whether associations between mother/father reports of negative interaction in the parent–adolescent relationship and early adolescent depressive symptoms varied as a function of adolescent reports of negative interaction. In other words, we tested for significant moderation effects. Based on theories that emphasize the importance of a fit between adolescents and their parents (e.g., Eccles et al. 1993; Lerner 1985; Thomas and Chess 1977), we expected significant interactions between adolescents’ and parents’ perceptions of their relationship in association with early adolescent depressive symptoms. Specifically, we hypothesized that larger discrepancies between adolescents’ and parents’ perceptions of their relationship would be associated with higher early adolescent depressive symptoms.

In addition, we tested whether the associations between adolescent and mother/father reports of negative interaction in the parent–adolescent relationship as well as interactions between adolescents’ and parents’ perceptions of their relationship and early adolescent depressive symptoms were moderated by adolescent gender. In line with interpersonal models of depression, which suggest that adolescent girls place greater value on their interpersonal relationships than do boys and that disturbances in these relationships may thus more strongly affect adolescent girls than boys (Rudolph 2009), we expected that, if we would find any significant gender differences, associations between (discrepancies in) negative interaction in the parent–adolescent relationship and early adolescent depressive symptoms would be stronger for early adolescent girls than boys.

## Method

### Participants

Participants in this two-wave longitudinal community study were 497 adolescents (57 % boys, *M*_age_ T_1_ = 13.03 years, *SD*_age_ = 0.46; range 11.01–15.56 years), their mothers (*n* = 497, *M*_age_ T_1_ = 44.41 years, *SD*_age_ = 4.45), and their fathers (*n* = 446, *M*_age_ T_1_ = 46.75 years, *SD*_age_ = 5.05). All adolescents identified themselves as ethnic Dutch and attended the first year of secondary school at the start of the study. The majority of adolescents lived in intact two-parent families (85.2 %) at the start of the study. Furthermore, based on parents’ job level, 10.8 % of the families were characterized by low SES. Data are part of young cohort of the Research on Adolescent Development and Relationships (RADAR) study in the Netherlands.

Sample attrition was low across the 1-year interval between waves, with 466 of the 497 adolescents (94 %), 462 of the 497 mothers (93 %), and 424 of the 446 fathers (95 %) participating at both measurement waves. Adolescents who participated at both waves reported slightly lower levels of depressive symptoms at the start of the study than those dropping out, *F*(1, 489) = 6.53, *p* = .01, *η*^2^ = .01, but there were no significant differences with respect to age, *F*(1, 495) = 0.10, *p* = .75, gender, χ^2^(1) = 0.60, *p* = .81, or reports of parent–adolescent relationship quality at the start of the study, *F*(2, 459) = 1.27, *p* = .28. Mothers and fathers still participating at the second annual measurement wave did not significantly differ from those dropping out of the study with respect to age, *F*(1, 495) = 1.84, *p* = .18 and *F*(1, 443) = 1.24, *p* = .27, respectively, or reports of parent–adolescent relationship quality at the start of the study, *F*(1, 492) = 3.49, *p* = .06 and *F*(1, 443) = 1.91, *p* = .17, respectively.

### Procedure

Adolescents were recruited from randomly selected schools in the western and central regions of the Netherlands. Before the start of the study in January 2006, adolescents and their parents received a complete description of the study and provided active written informed consent to participate. Adolescents and their parents were invited to complete annual questionnaires during a home visit. Participants received a small monetary compensation for every wave they completed the questionnaires. This study was approved by the board of the local research institute and the Medical Ethical Committee of the Utrecht Medical Centre in the Netherlands.

### Measures

#### Adolescent Depressive Symptoms

We used the Dutch version of the Reynolds Adolescent Depression Scale-second edition (RADS-2; Reynolds 2000) to assess adolescent depressive symptoms. The RADS-2 is a 23-item self-report questionnaire, with items being measured on a 4-point scale ranging from 1 (*almost never*) to 4 (*usually*). A sample item reads “I am sad”. In this study, internal consistency for this scale was found to be good across both waves, with Cronbach’s alpha ranging from .90 to .94. Higher scores indicate higher depressive symptoms.

#### Parent–Adolescent Relationship Quality

We used the 6-item negative interaction subscale of the shortened Dutch version of the Network of Relationships Inventory (NRI; Furman and Buhrmester 1985) to assess negative aspects of the mother–adolescent and father–adolescent relationship as perceived by adolescents, mothers, and fathers. All items were rated on a 5-point scale, ranging from 1 (*little or none*) to 5 (*the most*). A sample item reads “How much do you and your mother/father argue with each other?” for negative interaction perceived by the adolescent in the mother–adolescent and father–adolescent relationship, respectively. Higher scores indicate higher levels of negative interaction. In this study, internal consistency for the negative interaction subscale was found to be good across informants. Specifically, Cronbach’s alpha was .90 for adolescent-reports on the mother–adolescent relationship, .89 for adolescent-reports on the father–adolescent relationship, .92 for mother-reports on the mother–adolescent relationship, and .90 for father-reports on the father–adolescent relationship.

### Statistical Analyses

We conducted regression analyses including interaction terms (i.e., polynomial regression analyses) in M*plus* Version 7.4 to examine adolescent-parent discrepancies in reports of negative interaction in the mother–adolescent and the father–adolescent relationship using statistical tests of moderation between parent- and adolescent-reports. Such moderation analyses indicate whether the interaction between two informants’ reports provides any new information beyond the main effects of the individual informants’ reports, and test whether high (or low) scores from one informant are more or less strongly associated with the outcome depending on the other informant’s scores. Thereby, the use of an interaction term between mother’s/father’s and adolescent’s perception of the parent–adolescent relationship allows for a test for multiple patterns of informant discrepancies that cannot be captured using any of the different types of difference scores. All independent variables were mean-centered before analysis to increase interpretability of the regression estimates as well as reduce problems of multicollinearity.^1^ Our polynomial regression analyses included adolescent- and parent-reports of negative interaction, the interaction between adolescent- and parent-reports, and the squared adolescent- and parent-reports (as the interaction between adolescent and parent reports may reflect the squared effect of either of these reports if the squared effects are not included in the analyses). Significant interaction terms were interpreted by visualizing predicted values of early adolescent depressive symptoms and calculating simple slopes at low (−1 SD) and high (+1 SD) levels of adolescent-reported and parent-reported negative interaction (Aiken and West 1991).

All polynomial regression analyses were conducted concurrently as well as longitudinally over a 1-year period, controlling for stability in adolescent depressive symptoms by including 1-year earlier symptom levels as a predictor in the analyses. Furthermore, analyses were conducted separately for the mother–adolescent and father–adolescent relationship and were tested for potential moderation by adolescent gender.

Furthermore, solely to enable comparisons with previous studies relying on difference scores, we also calculated three types of difference scores regarding negative interaction in the mother–adolescent and the father–adolescent relationship, which are currently most widely used as indicators of informant discrepancies. Yet, we refrain from interpreting these findings because others have identified several issues that challenge the validity and interpretation of difference scores as indicators of informant discrepancies (Edwards 1994, 2002; Laird and De Los Reyes 2013). First, a directional difference score (*D*) was computed, by subtracting the mother- and father-reports from the adolescent-reports of negative interaction. This type of difference score supposedly captures mean-level differences between informants. Second, a squared difference score (*D*^2^) was computed, by squaring the directional difference scores. Compared to *D*, this type of difference score supposedly is a more sensitive indicator of the degree of discrepancy between informants, by squaring initial mean-level differences between informants. Third, a directional difference score in standardized reports (*DZ*) was computed by first standardizing the parent- and adolescent-reports of negative interaction before subtracting the mother- and father-reports from the adolescent-reports. This type of difference score supposedly captures mean-level differences between informant reports while controlling for absolute levels of the construct of interest (De Los Reyes and Kazdin 2004). In line with our polynomial regression analyses, we examined both concurrent and 1-year longitudinal associations between these difference scores and early adolescent depressive symptoms in separate regression analyses in M*plus* Version 7.4. Furthermore, analyses were conducted separately for the mother–adolescent and father–adolescent relationship and were tested for potential moderation by adolescent gender.

## Results

### Descriptive Statistics

Table 1 provides an overview of the means and standard deviations of all study variables as well as tests of congruency between parent and adolescent reports of negative interaction by paired *t* tests for mean-level differences and by bivariate correlations. Adolescents reported slightly higher levels of negative interaction than their mothers, but no significant differences were found in reports of negative interaction between adolescents and their fathers. Furthermore, adolescent and parent reports of negative interaction in the parent–adolescent relationship were significantly associated for both mothers and father, *r*s = .45 and .52, respectively.

**Table 1:** Means and SDs of all study variables as well as tests of congruency between parent and adolescent reports of negative interaction in the parent–adolescent relationship

Variable	Adolescent-report		Mother-report		Father-report		Δ Parent–adolescent report		*r* Parent–adolescent report
	*M*	*SD*	*M*	*SD*	*M*	*SD*	*M*	*t*	
T_1_ adolescent depression	37.55	11.34							
T_2_ adolescent depression	34.60	11.43							
T_1_ adolescent-mother NI	1.66	0.58	1.52	0.53			0.14	5.33*	.45*
T_1_ adolescent-father NI	1.51	0.56			1.51	0.50	0.01	0.37	.52*

*NI* negative interaction

* *p* < .001

Associations between separate reports of adolescents, mothers, and fathers on negative interaction in the parent–adolescent relationship and early adolescent depressive symptoms are reported in Table 2. The results suggest small-to-moderate positive associations between all reports of negative interaction and concurrent (T_1_) adolescent depressive symptoms. Yet, when we examined partial associations with adolescent depressive symptoms longitudinally (T_2_), while controlling for stability in depressive symptoms from T_1_ to T_2_, almost no significant associations were found (see Table 2). This pattern of findings suggests that negative interaction in the parent–adolescent relationship is not associated with changes in early adolescent depressive symptoms over a 1-year period when taking stability in adolescent depressive symptoms into account.

**Table 2:** Bivariate and partial associations between parent and adolescent reports of negative interaction and early adolescent depressive symptoms at T_1_ and T_2_

Informant	Concurrently (T_1_)	Longitudinally (T_2_)^a^
Adolescent-reported NI in mother–adolescent relationship	.33**	.04
Adolescent-reported NI in father–adolescent relationship	.32**	.07
Mother-reported NI	.26**	.12*
Father-reported NI	.16**	.01

*NI* negative interaction

* *p* ≤ .01; ** *p* ≤ .001

^a^Partial associations were estimated, controlling for adolescent depressive symptoms at T_1_

### Discrepancies in Reports of the Parent–Adolescent Relationship and Early Adolescent Depressive Symptoms

#### Polynomial Regression Analyses

**Fig. 1:**
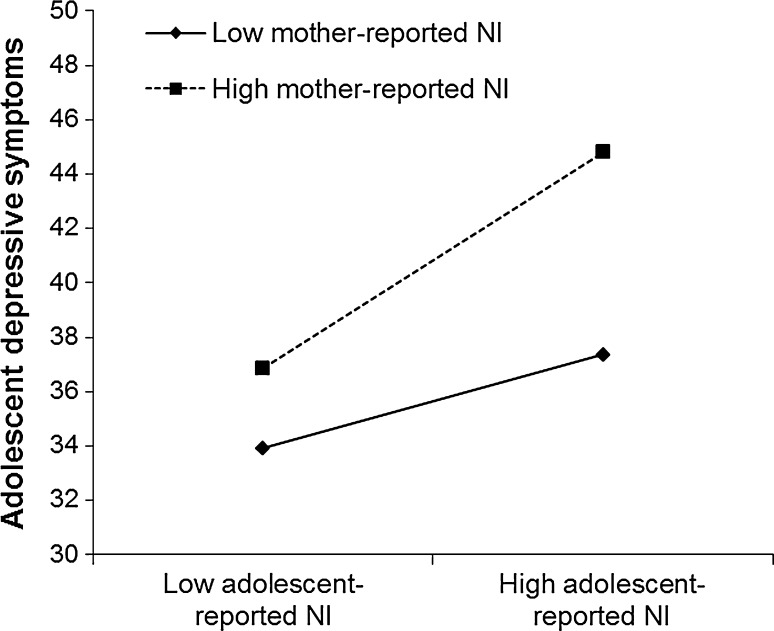
Early adolescent depressive symptoms at low (−1 SD) and high (+1 SD) levels of adolescent-reported and mother-reported NI. *NI* negative interaction. Adolescent depressive symptom scores ranged from 23 to 92

**Fig. 2:**
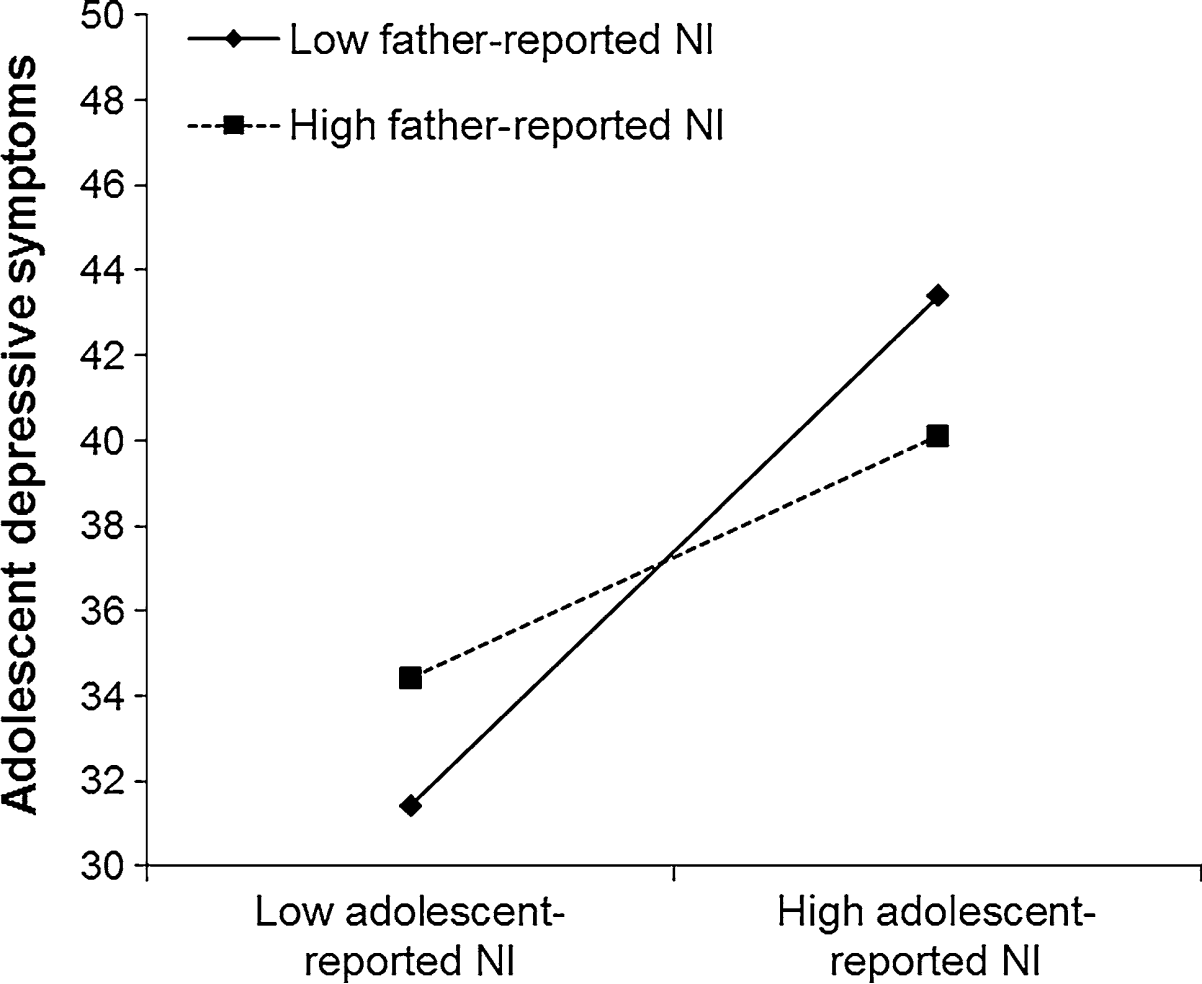
Early adolescent depressive symptoms at low (−1 SD) and high (+1 SD) levels of adolescent-reported and father-reported NI. *NI* negative interaction. Adolescent depressive symptom scores ranged from 23 to 92

Results from the polynomial regression analyses are reported in Table 3. Most important for this study, the interaction term between parent-reported and adolescent-reported negative interaction in the parent–adolescent relationship was significantly associated with concurrent (T_1_) adolescent depressive symptoms for both the mother–adolescent (β = .16) and the father–adolescent relationship (β = −.29). Visualization of these significant interaction terms suggested interesting differences between reports of the mother–adolescent and father–adolescent relationship in association with early adolescent depressive symptoms (see Figs. 1 and 2).

**Table 3:** Polynomial regression analyses predicting early adolescent depressive symptoms

Predictor	Concurrently (T_1_)			Longitudinally (T_2_)^a^		
	*b*	SE	β	*b*	SE	β
Adolescent report NI	4.942***	1.129	.251	0.404	1.062	.020
Mother report NI	4.904***	1.271	.229	3.772**	1.198	.175
(Adolescent report NI)^2^	−0.545	1.049	−.036	−0.253	0.957	−.017
(Mother report NI)^2^	−3.873**	1.265	−.218	−0.970	1.154	−.056
Adolescent × mother report NI	3.701*	1.752	.164	−1.298	1.588	−.058
Adolescent depressive symptoms T_1_				0.547***	0.042	.534
Model *R* ^2^ (*n*)	.14 (*n* = 487)			.34 (*n* = 458)		
Adolescent report NI	7.876***	1.359	.395	2.478	1.280	.125
Father report NI	−0.160	1.412	−.007	0.268	1.309	.012
(Adolescent report NI)^2^	0.323	1.485	.024	−1.480	1.350	−.112
(Father report NI)^2^	2.869	1.850	.141	−2.552	1.687	−.128
Adolescent × father report NI	−5.631*	2.445	−.292	2.490	2.230	.131
Adolescent depressive symptoms T_1_				0.536***	0.044	.526
Model *R* ^2^ (*n*)	.13 (*n* = 441)			.31 (*n* = 421)		

*NI* negative interaction

* *p* ≤ .05; ** *p* ≤ .01; *** *p* ≤ .001

^a^Controlling for adolescent depressive symptoms at T_1_

Specifically, results suggested that adolescent-reported negative interaction was more strongly associated with more adolescent depressive symptoms at *high* levels of mother-reported negative interaction, *b* = 8.64, *p* < .001, than at *low* levels of mother-reported negative interaction, *b* = 1.24, *p* = .54. These results indicated that the congruence of low mother-reported and low adolescent-reported negative interaction was associated with the lowest levels of adolescent depressive symptoms, whereas congruence of high mother-reported and adolescent-reported negative interaction was associated with the highest levels of adolescent depressive symptoms (see Fig. 1). In contrast, adolescent-reported negative interaction was more strongly associated with more adolescent depressive symptoms at *low* levels of father-reported negative interaction, *b* = 13.51, *p* < .001, than at *high* levels of father-reported negative interaction, *b* = 2.25, *p* = .47. These results indicated that the discrepancy of low father-reported negative interaction and high adolescent-reported negative interaction was associated with the highest levels of adolescent depressive symptoms, whereas congruence of low father-reported and low adolescent-reported negative interaction was associated with the lowest levels of adolescent depressive symptoms (see Fig. 2).

Importantly, controlling for stability in depressive symptoms from T_1_ to T_2_, β = .54–.55, *p* < .001, none of the interaction terms between parent-reported and adolescent-reported negative interaction were significant (see Table 3). These findings suggest that the interaction between parent and adolescent views of negative interaction in the parent–adolescent relationship does not have any additional predictive value above-and-beyond the relative stability of early adolescent depressive symptoms over a 1-year period. Finally, none of the effects of parent-reported negative interaction, adolescent-reported negative interaction, nor the interaction between parent-reported and adolescent-reported negative interaction on concurrent (T_1_) or longitudinal (T_2_) adolescent depressive symptoms, in either the mother–adolescent or the father–adolescent relationship, were significantly moderated by adolescents’ gender or family constellation (i.e., intact 2-parent families vs. other family constellations^2^).

#### Difference Score Analyses (to Enable Comparisons with Previous Studies)

Table 4 includes associations between the three types of difference scores, that is, directional, squared, and standardized, and early adolescent depressive symptoms. Concurrently, results suggested that difference scores with respect to father and adolescent reports of negative interaction were more strongly associated with early adolescent depressive symptoms at T_1_ (β = .16–.22) than difference scores with respect to mother and adolescent reports of negative interaction (β = .07–.09). Yet, we found no significant partial associations with adolescent depressive symptoms longitudinally (T_2_) when controlling for stability in depressive symptoms from T_1_ to T_2_, which suggests that none of the difference scores was associated with changes in early adolescent depressive symptoms over a 1-year period.

**Table 4:** Bivariate and partial associations between difference scores with respect to adolescent and parent reports of negative interaction and early adolescent depressive symptoms at T_1_ and T_2_

Difference scores	Concurrently (T_1_)	Longitudinally (T_2_)^a^
NI in mother–adolescent relationship		
*D*	.09*	−.06
*DZ*	.07	−.07
*D* ^2^	.08	.02
NI in father–adolescent relationship		
*D*	.19**	.06
*DZ*	.16**	.06
*D* ^2^	.22**	−.02

*NI* negative interaction, *D* directional difference score, *DZ* directional difference score in standardized reports, *D* ^2^ Squared directional difference score

* *p* ≤ .05; ** *p* ≤ .001

^a^Partial associations were estimated, controlling for adolescent depressive symptoms at T_1_

Most of the concurrent or longitudinal difference score associations in either the mother–adolescent or the father–adolescent relationship were not significantly moderated by adolescents’ gender or family constellation (i.e., intact 2-parent families vs. other family constellations). The sole exception to the general pattern for adolescent gender involved a significant moderation by adolescent gender of the association between the squared difference score with respect to the father–adolescent relationship and concurrent (T_1_) early adolescent depressive symptoms (*p* = .04), suggesting stronger effects for girls, β = .31, *p* < .001, than boys, β = .14, *p* = .03. However, this interaction effect was not significant longitudinally (T_2_) when controlling for stability in depressive symptoms from T_1_ to T_2_ (*p* = .96). The sole exception to the general pattern for family constellation involved a significant moderation by family constellation of the association between the squared difference score with respect to the mother–adolescent relationship and concurrent (T_1_) early adolescent depressive symptoms (*p* = .03), suggesting significant effects only for family constellations other than intact 2-parent families, β = .28, *p* = .01, and not for intact 2-parent families, β = .07, *p* = .17. Again, this interaction effect was not significant longitudinally (T_2_) when controlling for stability in depressive symptoms from T_1_ to T_2_ (*p* = .15).

## Discussion

Adolescence is a critical period for the development of depressive symptoms (e.g., Holsen et al. 2000; Kessler et al. 2012) and problems in the parent–adolescent relationship have been consistently associated with higher adolescent depressive symptoms (e.g., Hale et al. 2008; McLeod et al. 2007; Nelemans et al. 2014; Rudolph 2009). Yet, adolescents and parents often perceive their relationship quite differently and differences in perceptions of the parent–adolescent relationship, even more so than the perceptions themselves, may constitute a risk for adolescent depressive symptoms. According to goodness of fit models (e.g., Eccles et al. 1993; Lerner 1985), this may be especially the case in early adolescence. Therefore, in the present study we examined how discrepancies in adolescents’ and both mothers’ and fathers’ perceptions of the parent–adolescent relationship were associated with early adolescent depressive symptoms, concurrently as well as longitudinally over a 1-year period.

We followed recent analytical suggestions (Laird and De Los Reyes 2013; Laird and Weems 2011; in line with Edwards 1994, 2002) by using polynomial regression analyses including tests of moderation (i.e., including interaction terms between different informants’ perceptions), which have been proposed to represent more valid tests of hypotheses involving informant discrepancies than difference scores. Results suggested the highest levels of adolescent depressive symptoms with *congruence* in reports of high negative interaction between mothers and adolescents (Fig. 1), but also with *discrepancies* between adolescent-reported high negative interaction and father-reported low negative interaction (Fig. 2). This pattern of findings underscores the need for a more sophisticated methodology such as polynomial regression analysis including tests of moderation that can adequately address both congruence and discrepancies in perceptions of adolescents and mothers/fathers of the parent–adolescent relationship in detail. Such an analysis can contribute to a more comprehensive understanding of risk factors for early adolescent depressive symptoms.

### Discrepancies in Reports of the Parent–Adolescent Relationship and Early Adolescent Depressive Symptoms

Results from polynomial regression analyses including interaction terms between different informants’ perceptions suggested the highest levels of concurrent adolescent depressive symptoms when there was congruence in mother and adolescent reports of high negative interaction (see Fig. 1). These results appear to be partly in line with previous findings regarding conflict in the mother–adolescent relationship (Laird and De Los Reyes 2013), in a way that congruence of low negative interaction and conflict reported by both adolescents and mothers appears to be associated with the lowest levels of adolescent depressive symptoms. Yet, our findings seem to be more in line with principles of cumulative or additive risk models, by suggesting that congruence of high negative interaction reported by both adolescents and mothers appears to be associated with the highest levels of adolescent depressive symptoms. Intuitively, these results appear to be in contrast with goodness of fit models, from which we would expect that congruence between adolescents’ and mothers’ perceptions of their relationship may be reflective of better fit between them and may thereby be associated with lower adolescent depressive symptoms. Yet, agreement between mothers and adolescents that their relationship is characterized by high levels of conflict may still reflect a misfit in the mother–adolescent relationship, and may therefore be associated with high levels of early adolescent depressive symptoms.

Furthermore, results from polynomial regression analyses including interaction terms between different informants’ perceptions suggested the highest levels of concurrent adolescent depressive symptoms when there was a discrepancy between adolescent-reported high negative interaction and father-reported low negative interaction (see Fig. 2). These results are in line with previous findings using difference scores, which have suggested that (larger) informant discrepancies are associated with higher adolescent internalizing problems (e.g., Guion et al. 2009; Juang et al. 2007; Laird and De Los Reyes 2013; Sher-Censor et al. 2011). Moreover, these results are in line with suggestions in theoretical models on goodness of fit (Eccles et al. 1993; Lerner 1985; Lerner et al. 1986; Thomas and Chess 1977), which stress the importance of person-environment fit in a way that lower discrepancies between adolescents’ and parents’ perceptions of their relationship may be reflective of better fit between them and may thereby be associated with healthy adolescent development. In contrast, if adolescents’ characteristics, or needs, and parents’ demands do not match, this lack of fit may result in discrepancies between adolescents’ and parents’ perceptions of their relationship, and this reflection of incompatibility between adolescents and parents may be associated with higher adolescent depressive symptoms. Also, these findings suggest that when early adolescents experience levels of negative interaction that are not experienced at a comparable level by the parent, thereby implying a loss of the parent–adolescent bond, this is associated with adolescent depressive symptoms. Alternatively, results could imply that adolescents with high depressive symptoms have a distorted view of the parent–adolescent relationship and therefore report higher levels of negative interaction, which in turn are associated with adolescent depressive symptoms (i.e., *depression*-*distortion hypothesis;* De Los Reyes and Kazdin 2005).

Importantly, our results from polynomial regression analyses provide a much more nuanced picture regarding discrepancies between adolescents’ and fathers’ perceptions of their relationship and early adolescent depressive symptoms than previous studies relying on difference scores. For example, our results also suggested that adolescents who do not share their father’s negative view of the parent–adolescent relationship are somewhat “protected” with respect to depressive symptoms (in line with findings regarding adolescent and parent reports of the family in association with adolescent anxiety; Ohannessian and De Los Reyes 2014). These findings may suggest that experiences of paternal neglect may be associated with higher adolescent depressive symptoms.

### Informant Discrepancies: Polynomial Regression Analyses Versus Difference Scores

Recently, Laird and De Los Reyes (2013) have argued that discrepancies between informant reports may be more accurately captured by interaction terms in polynomial regression analysis rather than the use of difference scores (in line with Edwards 1994, 2002). The use of difference scores has been heavily criticized from both statistical and methodological points of view as well as with regard to the validity of these scores as indicators of informant discrepancies (e.g., Edwards 1994, 2002; Laird and De Los Reyes 2013; Laird and Weems 2011). Recent direct tests (Laird and De Los Reyes 2013) of constraints imposed by difference scores (Edwards 1994, 2002) have also suggested that conditions required for the use of directional or squared difference scores were never fully met. These results have demonstrated that difference scores cannot validly represent informant discrepancies, whereas interaction terms have shown criterion validity as reflections of parent–adolescent discrepancies and provide a more comprehensive and accurate test of informant discrepancies (De Los Reyes et al. 2013).

Studies from Laird and De Los Reyes (2013) and Tackett et al. (2013) have illustrated that the particular method selected to analyze informant discrepancies may substantially affect empirical conclusions regarding the risk of these discrepancies for adolescent adjustment. Polynomial regression analyses including tests of moderation between different informants’ perceptions are able to illustrate a more nuanced picture of associations between informant discrepancies and early adolescent depressive symptoms than results from difference scores. Hence, the use of such polynomial regression analyses as test of hypotheses involving informant discrepancies is recommended for future research (De Los Reyes et al. 2013).

### Strengths, Limitations, and Future Research

The present study makes a substantial contribution to the understanding of risks in the parent–adolescent relationship for early adolescent depressive symptom development. Such understanding is particularly salient in this developmental period for three major reasons. First, early adolescence is characterized by pronounced changes in the parent–adolescent relationship (Laursen and Collins 2009), which make discrepant perceptions between adolescents and parents regarding their relationship particularly likely to happen. Second, early adolescence is a period that is critical for the development of depressive symptoms (Cole et al. 2002; Rudolph 2009). Third, theoretical goodness of fit models emphasize that decreased person-environment fit is especially likely in the early adolescent developmental period (e.g., Eccles et al. 1993; Lerner 1985). Furthermore, by including both mothers and fathers in our study, we addressed the relative ignorance of the father–adolescent relationship in the current literature and we were able to examine the potential differential associations of informant discrepancies in the mother–adolescent and the father–adolescent family subsystems in association with adolescent depressive symptoms. Also, by examining all associations both concurrently and longitudinally over a 1-year period, while controlling for adolescents’ earlier symptoms of depression, we were able to examine if discrepancies in perceptions of the parent–adolescent relationship have any additional value above-and-beyond the relative stability of adolescent depressive symptoms over time. This longitudinal approach is important for future research to consider, given the relative stability of depressive symptoms over time (Holsen et al. 2000; Kessler et al. 2012). Finally, this study adds to the scarce literature including polynomial regression analyses as tests of hypotheses involving informant discrepancies, which have recently been put forward as more accurate and valid test of informant discrepancies than the use of difference scores (De Los Reyes et al. 2013; Edwards 1994, 2002; Laird and De Los Reyes 2013), in the field of parent–adolescent discrepancies and adolescent depressive symptoms in particular.

Yet, some limitations of this study should also be noted. First, our sample was fairly homogeneous, consisting largely of medium-to-high SES, intact two-parent Dutch families. So caution should be exercised when generalizing our findings. Future research may want to rely on samples that are more diverse in terms of both ethnicity and family constellation (e.g., divorced co-parents, single-parent) to examine the generalizability of our findings to different situations. Second, we focused on only one key dimension of parent–adolescent relationship quality, that is, the negative, conflictual aspect, which has been consistently associated with adolescent depressive symptoms (e.g., Hale et al. 2008; McLeod et al. 2007; Nelemans et al. 2014). Also, interpersonal difficulties in the parent–adolescent relationship are central to several theories on adolescent depression (e.g., Joiner and Coyne 1999; Rudolph 2009). However, meta-analytic results (McLeod et al. 2007) have suggested that other (sub)dimensions of the parent–adolescent relationship are also associated with adolescent depressive symptoms, such as parental warmth or overinvolvement, which may be important to consider in future research using polynomial regression analyses to examine associations between parent–adolescent discrepancies in views of the parent–adolescent relationship and adolescent depressive symptoms. Third, we used a unidirectional approach to the study of associations between informant-discrepancies and adolescent depressive symptoms, by focusing on how discrepant views of the parent–adolescent relationship may predict adolescent depressive symptoms (i.e., a so-called *parent*-*effects* model; Branje et al. 2008; Lollis and Kuczynski 1997). Yet, several theories stress that adolescent depressive symptoms also affect the parent–adolescent relationship (Joiner and Coyne 1999; Rudolph 2009) and bidirectional associations are increasingly supported by empirical findings (e.g., Hale et al. 2008; Nelemans et al. 2014). Thus, in future research it would be important to not only consider informant discrepancy as potential predictor of adolescent adjustment, but as potential outcome of adolescent adjustment as well (depression–distortion hypothesis; De Los Reyes and Kazdin 2005).

For future research it is also important to consider how our conclusions regarding associations between parent–adolescent discrepancies and early adolescent depressive symptoms were affected by examining these associations concurrently as well as longitudinally over a 1-year period, while controlling for adolescents’ earlier symptoms of depression. Specifically, we found that parent–adolescent congruence or discrepancies were not significantly associated with changes in adolescent depressive symptoms over a 1-year period, above-and-beyond the relative stability of early adolescent depressive symptoms. Associations thus appear to be more short-term than long-term, or more associated with concurrent depressive symptom levels rather than changes in depressive symptoms over a 1-year period.

Furthermore, our findings suggested that discrepancies in parents’ and adolescents’ reports of the father–adolescent relationship were more strongly associated with early adolescent depressive symptoms than discrepancies in reports of the mother–adolescent relationship, which is in line with some previous findings (e.g., Sher-Censor et al. 2011). Perhaps discrepancies in mother–adolescent relationship are not necessarily unhealthy, but may rather reflect normative developmental processes associated with individuation and adolescents’ striving for greater autonomy (De Goede et al. 2009; Laursen and Collins 2009), especially during early adolescence. This interpretation appears to be supported by some findings in our study, including descriptive findings that adolescents and their mothers differed much more in their perception of the parent–adolescent relationship than adolescents and their fathers (Table 1). By contrast, discrepancies in father–adolescent relationship seem unhealthier. However, few studies have included fathers (and their perceptions of the family or parent–adolescent relationship) and findings regarding the relative importance of fathers and mothers for adolescent depressive symptoms are inconsistent (e.g., Brumariu and Kerns 2010). So although there is too little research to draw any conclusions from, the results in the present study argue for distinguishing between relationships with mothers and fathers in future research. This recommendation is in line with family systems theory (Minuchin 1985; Restifo and Bögels 2009), which argues that the mother–adolescent and father–adolescent relationships represent distinct but related subsystems within the family, in which different aspects of quality may be differentially associated with adolescent depressive symptoms. Also, from a family systems perspective, it may be interesting for future research to examine how discrepancies in the mother–adolescent and father–adolescent relationship might interact in predicting adolescent depressive symptoms.

Another interesting approach for future research on informant discrepancies would be to combine polynomial regression analysis with response surface analysis (Shanock et al. 2010). This approach would allow researchers to graph the results of polynomial regression analyses in a three-dimensional—rather than a two-dimensional—figure. In such a three-dimensional space, conclusions regarding informant agreement, degree of discrepancy, and direction of the discrepancy and an outcome variable are represented as a continuous surface, supported by statistical test of significance of different surface values. Through these additional features, response surface analysis further increases the explanatory potential of polynomial regression analyses.

## Conclusion

In this study, both congruence in adolescents’ and mothers’ perceptions of a highly conflictual mother–adolescent relationship and discrepancies in adolescents’ and fathers’ perceptions of the father–adolescent relationship were found to be associated with higher early adolescent depressive symptoms. These associations were stronger for concurrent depressive symptoms than changes in symptoms over a 1-year period, controlling for stability in adolescent depressive symptoms over this period of time. Results from our polynomial regression analyses, which have been suggested as more accurate and valid reflections of informant discrepancies than the use of difference scores (Edwards 1994, 2002; Laird and De Los Reyes 2013; Laird and Weems 2011), are able to illustrate a more nuanced picture of associations between informant discrepancies and early adolescent depressive symptoms than results from difference score analyses. Hence, the use of polynomial regression analyses as test of hypotheses involving informant discrepancies may be recommended for future research.

## Footnotes

1 Exploratory analyses suggested that multicollinearity was not likely a problem in our polynomial regression analyses. Specifically, although the correlations between the squared and multiplicative interaction terms and the original variables from which they were constructed were relatively high across our different models, *r*s = .41–.69, these correlations are not so high that they may be expected to distort results. Moreover, the variance inflation factor (VIF) values of all variables across all models were below the most commonly used rule of thumb of 10.

2 Post-hoc analyses were conducted for possible moderation of all associations between parents’ and adolescents’ reports of the parent–adolescent relationship and early adolescent depressive symptoms by family constellation.
